# The Relationship between the SARC-F Score and the Controlling Nutritional Status Score in Gastrointestinal Diseases

**DOI:** 10.3390/jcm11030582

**Published:** 2022-01-24

**Authors:** Takako Ikegami, Hiroki Nishikawa, Masahiro Goto, Masahiro Matsui, Akira Asai, Kosuke Ushiro, Takeshi Ogura, Toshihisa Takeuchi, Shiro Nakamura, Kazuki Kakimoto, Takako Miyazaki, Shinya Fukunishi, Hideko Ohama, Keisuke Yokohama, Hidetaka Yasuoka, Kazuhide Higuchi

**Affiliations:** 1 The Second Department of Internal Medicine, Osaka Medical and Pharmaceutical University, Takatsuki 569-8686, Osaka, Japan; takako.ikegami@ompu.ac.jp (T.I.); masahiro.goto@ompu.ac.jp (M.G.); masahiro.matsui@ompu.ac.jp (M.M.); akira.asai@ompu.ac.jp (A.A.); ushiro.1989@icloud.com (K.U.); oguratakeshi0411@yahoo.co.jp (T.O.); toshihisa.takeuchi@ompu.ac.jp (T.T.); shiro.nakamura@ompu.ac.jp (S.N.); kazuki.kakimoto@ompu.ac.jp (K.K.); takako.miyazaki@ompu.ac.jp (T.M.); shinya.fukunishi@ompu.ac.jp (S.F.); hideko.ohama@ompu.ac.jp (H.O.); hammer_0906@yahoo.co.jp (K.Y.); hidetaka.yasuoka@ompu.ac.jp (H.Y.); kazuhide.higuchi@ompu.ac.jp (K.H.); 2 The Premier Departmental Research of Medicine, Osaka Medical and Pharmaceutical University, Takatsuki 569-8686, Osaka, Japan

**Keywords:** SARC-F, CONUT, sarcopenia, malnutrition, gastrointestinal disease

## Abstract

We sought to examine the relationship between the SARC-F score and the Controlling Nutritional Status (CONUT) score in patients with gastrointestinal diseases (GDs, *n* = 735, median age = 71 years, and 188 advanced cancer cases). The SARC-F score ≥ 4 (highly suspicious of sarcopenia) was found in 93 cases (12.7%). Mild malnutritional condition was seen in 310 cases (42.2%), moderate in 127 (17.3%) and severe in 27 (3.7%). The median SARC-F scores in categories of normal, mild, moderate and severe malnutritional condition were 0, 0, 1 and 1 (overall *p* < 0.0001). The percentage of SARC-F score ≥ 4 in categories of normal, mild, moderate and severe malnutritional condition were 4.4%, 12.9%, 26.8% and 25.9% (overall *p* < 0.0001). The SARC-F score was an independent factor for both the CONUT score ≥ 2 (mild, moderate or severe malnutrition) and ≥5 (moderate or severe malnutrition). In the receiver operating characteristic (ROC) curve analysis for the CONUT score ≥ 2, C reactive protein (CRP) had the highest area under the ROC (AUC = 0.70), followed by the SARC-F score (AUC = 0.60). In the ROC analysis for the CONUT score ≥ 5, CRP had the highest AUC (AUC = 0.79), followed by the SARC-F score (AUC = 0.63). In conclusion, the SARC-F score in patients with GDs can reflect malnutritional status.

## 1. Introduction

Sarcopenia is characterized by generalized loss of muscle mass and muscle functional decline, resulting in physical frailty, cachexia and mortality [1,2]. Malnutrition, reticence, advanced malignancy-bearing status and persistent inflammatory status frequently observed in gastrointestinal diseases (GDs) are representative features associated with sarcopenia [1,2,3,4,5,6,7,8,9]. Reduced daily dietary intakes and deterioration of nutritional status can be also often seen in patients with GDs [8], and sarcopenia in patients with GDs is associated with poorer patient quality of life (QOL) and prognosis [8,10]. GD is a prime example of secondary sarcopenia due to the disease burden [10]. Nishigori, et al. reported that out of 199 patients with esophageal cancer, 149 patients (75%) had sarcopenia [11]. Huang, et al. reported that out of 173 patients with gastric cancer, 52 (30%) had sarcopenia [12]. In patients with colon cancer, 39–48% patients have been reported to involve sarcopenia [13,14]. In patients with pancreatic cancer, approximately 60% have been reported to have sarcopenia [15]. In patients with hepatocellular carcinoma, 11–66% have been reported to have sarcopenia [6].

SARC-F (strength (S), assistance walking (A), rising from a chair (R), climbing stairs (C), and falls (F)) is a questionnaire with five questions for the purpose of screening for sarcopenia [16,17]. Subjects are asked to reply to the five questions on a scale of 0 to 2, and the sum is calculated [16]. Subjects with a SARC-F score ≥ 4 are considered to be highly suspicious cases for sarcopenia [16]. The SARC-F score can be linked to physical functional reserve and QOL [18,19]. SARC-F is recommended to be used as an initial screening method for sarcopenia in the current international guidelines [20,21], whereas its lower sensitivity for sarcopenia may be a problem [22,23,24]. On the other hand, the controlling nutritional status (CONUT) score is a nutritional assessment tool, reported in 2002, in which serum albumin value, peripheral blood lymphocyte count, and total cholesterol value are scored and a total score is calculated [25,26]. The level of malnutrition is evaluated according to four levels: normal, mild, moderate, and severe. The higher the score, the more severe the malnutrition [25,26]. The CONUT score has the advantage of being simple and quick to calculate. The CONUT is a well validated screening tool for malnutrition and it correlates well with patient-generated subjective global assessment [26].

To our knowledge, however, there have been no reports regarding the relationship between the SARC-F score and the CONUT score in patients with GDs. The SARC-F does not include questions about nutrition. These issues deserve to be addressed, which urged us to conduct the current study.

## 2. Patients and Methods

### 2.1. Patients and Our Study

In the Second Department of Internal Medicine of the Osaka Medical and Pharmaceutical University (OMPU) Hospital, sarcopenia risk was assessed using the SARC-F. As a rule, all hospitalized patients were asked to answer the SARC-F questionnaire on admission. Between May 2020 and April 2021, 735 Japanese GD patients with both the SARC-F score and the CONUT score could be found in our database. As described above, the SARC-F score and the CONUT score for each patient was evaluated. First, the SARC-F score and the percentage of patients with a SARC-F score ≥ 4 were compared based on the nutritional condition as evaluated by the CONUT score. Next, univariate and multivariate analyses for the malnutrition were performed. Finally, receiver operating characteristic curve (ROC) analysis for the malnutrition, etc. was performed. Cancer of Stage III or worse was defined as advanced cancer. The ethics committee of OMPU hospital provided ethical approval (approval number, 2021-095).

### 2.2. The CONUT Score

The CONUT score involves three variables; total peripheral lymphocyte count, serum albumin value, and total cholesterol value [25,26]. A score of 0 was given for a total lymphocyte count of ≥1600 /μL, 1 for ≥1200 and <1600 /μL, 2 for ≥800 and <1200 /μL, and 3 for <800 /μL. A score of 0 was given for serum albumin value ≥3.5 g/dL, 2 for ≥3.0 and <3.5 g/dL, 4 for ≥2.5 and <3.0 g/dL, and 6 for <2.5 g/dL. A score of 0 was given for a total cholesterol value of ≥180 mg/dL, 1 for ≥140 and <180 mg/dL, 2 for ≥100 and <140 mg/dL, and 3 for <100 mg/dL. The total score (i.e., the CONUT score) was calculated. According to the CONUT score, study subjects were divided into four categories: (1) normal nutritional condition (0 or 1 point); (2) mild malnutritional condition (2, 3 or 4 points); (3) moderate malnutritional condition (5, 6, 7 or 8 points) and (4) severe malnutritional condition (9, 10, 11, 12 points) [25,26].

### 2.3. Statistics

In the analysis of continuous variables, the appropriate choice of Student’s *t* test, Mann-Whitney *U* or the and Pearson correlation coefficient (*r*) was made to compare the two groups, and the appropriate choice between the ANOVA and Kruskal-Wallis tests was made to compare multiple groups. Continuous variables were shown as median (interquartile range, IQR). In the analysis of categorical variables, the Pearson χ^2^ test was adopted to evaluate between-group difference. Multivariate logistic regression analysis related to the CONUT score ≥ 2 or the CONUT score ≥ 5 was done to extract independent variables using significant factors in the univariate analysis. Multivariate analysis using a cumulative logistic model was also performed. ROC analysis was done for the area under the ROC (AUC) and sensitivity/specificity, and the cutoff was adopted as the point where the sum of sensitivity and specificity is maximized. *p* = 0.05 was set at the significant level by the JMP ver. 15 (SAS Institute Inc., Cary, NC, USA). For the comparison between each group of two in multiple-group comparisons, the Bonferroni correction was adopted for adjusting type I error.

## 3. Results

### 3.1. Patient Baseline Features

Baseline features for all study subjects (*n* = 735, 438 males and 297 females, median (IQR) age = 71 (62–77) years) are shown in Table 1. The median (IQR) body mass index (BMI) was 22.2 (19.7–24.5) kg/m^2^. Upper gastrointestinal disease (UGD) was seen in 234 patients (advanced cancer, 66 (28.2%)), lower gastrointestinal disease (LGD) in 190 (advanced cancer, 41 (21.6%)), biliary and pancreatic disease (BPD) in 176 (advanced cancer, 46 (26.1%)) and liver disease (LD) in 135 (advanced cancer, 35 (25.9%)). Overall, advanced cancer was identified in 188 patients (25.6%). Details of diagnosis names are shown in Supplementary Table S1. SARC-F score 0 was found in 471 cases (64.1%), 1 in 89 (12.1%), 2 in 50 (6.8%), 3 in 32 (4.4%) and ≥4 in 93 (12.7%). Normal nutritional condition was found in 271 cases (36.9%), mild malnutritional condition in 310 cases (42.2%), moderate malnutritional condition in 127 cases (17.3%), and severe malnutritional condition in 27 cases (3.7%).

### 3.2. SARC-F Score Based on the Nutritional Condition

**Figure 1:**
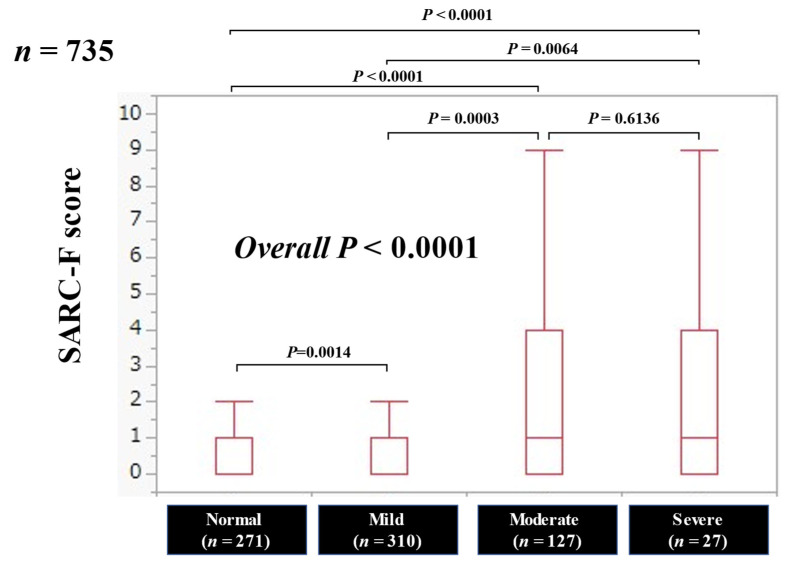
The SARC-F score based on the nutritional condition.

The SARC-F score significantly correlated with the CONUT score (*r* = 0.26, *p* < 0.0001). The median (IQR) SARC-F scores in categories of normal, mild, moderate and severe malnutritional condition were: 0 (0–1) in the normal, 0 (0–1) in the mild, 1 (0–4) in the moderate and 1 (0–4) in the severe, respectively, (*p* = 0.0014 (normal versus mild), *p* < 0.0001 (normal versus moderate), *p* < 0.0001 (normal versus severe), *p* = 0.0003 (mild versus moderate), *p* = 0.0064 (mild versus severe), *p* = 0.6136 (moderate versus severe), and overall *p* < 0.0001) (Figure 1).

### 3.3. The Percentage of Subjects with SARC-F Score ≥ 4 Based on the Nutritional Condition

**Figure 2:**
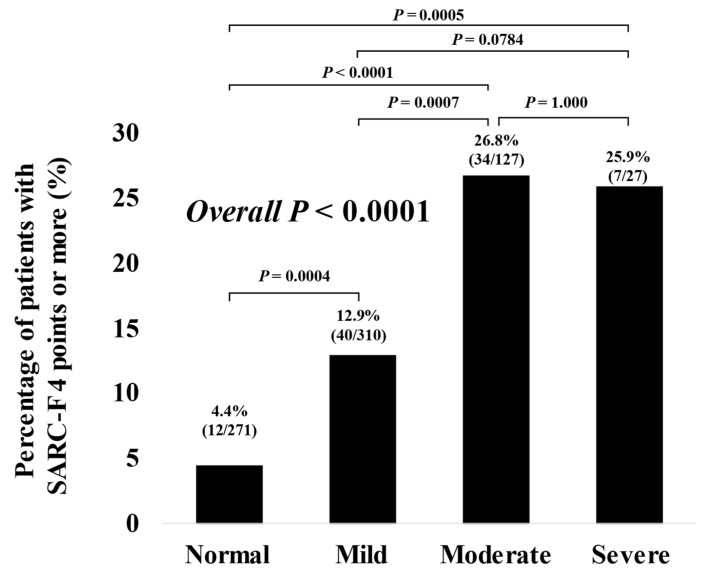
The percentage of patients with SARC-F score ≥ 4 based on the nutritional condition.

The percentage of SARC-F score ≥ 4 in categories of normal, mild, moderate and severe malnutritional condition were: 4.4% (12/271) in the normal, 12.9% (40/310) in the mild, 26.8% (34/127) in the moderate and 25.9% (7/127) in the severe, respectively, (*p* = 0.0004 (normal versus mild), *p* < 0.0001 (normal versus moderate), *p* = 0.0005 (normal versus severe), *p* = 0.0007 (mild versus moderate), *p* = 0.0784 (mild versus severe), *p* = 1.000 (moderate versus severe), and overall *p* < 0.0001) (Figure 2).

### 3.4. SARC-F Score Based on the Nutritional Condition in Subjects with and without Advanced Cancer

**Figure 3:**
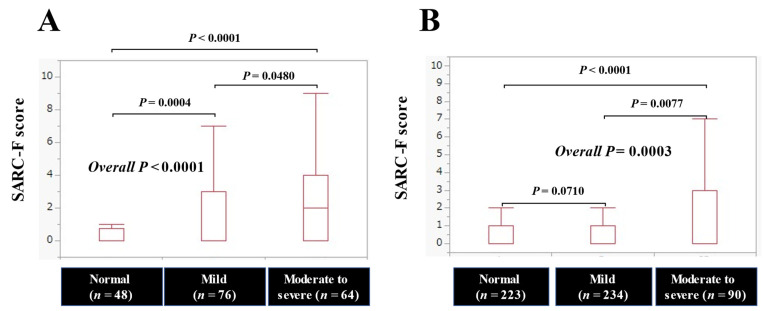
The SARC-F score based on the nutritional condition in patients with (**A**) and without (**B**) advanced cancer.

The median (IQR) SARC-F scores in categories of normal condition, mild malnutritional condition, and moderate to severe malnutritional condition in patients with advanced cancer (*n* = 188) were: 0 (0–0.75) in the normal (*n* = 48), 0 (0–3) in the mild (*n* = 76), and 2 (0–4) in the moderate to severe (*n* = 64), respectively, (*p* = 0.0004 (normal versus mild), *p* = 0.0480 (mild versus moderate to severe), *p* < 0.0001 (normal versus moderate to severe), and overall *p* < 0.0001). (Figure 3A)

The median (IQR) SARC-F scores in categories of normal condition, mild malnutritional condition, and moderate to severe malnutritional condition in patients without advanced cancer (*n* = 547) were: 0 (0–1) in the normal (*n* = 223), 0 (0–1) in the mild (*n* = 234), and 0 (0–3) in the moderate to severe (*n* = 90), respectively, (*p* = 0.0710 (normal versus mild), *p* = 0.0077 (mild versus moderate to severe), *p* < 0.0001 (normal versus moderate to severe), and overall *p* = 0.0003). (Figure 3B)

### 3.5. The Percentage of Subjects with SARC-F Score ≥ 4 Based on the Nutritional Condition in Subjects with and without Advanced Cancer

**Figure 4:**
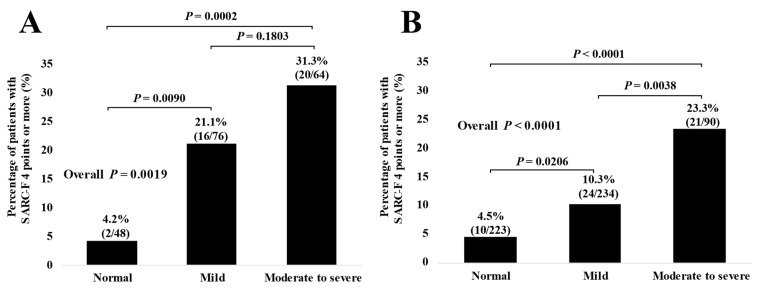
The percentage of patients with SARC-F score ≥ 4 based on the nutritional condition in patients with (**A**) and without (**B**) advanced cancer.

The percentage of SARC-F score ≥ 4 in categories of normal condition, mild malnutritional condition, and moderate to severe malnutritional condition in patients with advanced cancer were: 4.2% (2/48) in the normal, 21.1% (16/76) in the mild, and 31.3% (20/64) in the moderate to severe, respectively, (*p* = 0.0090 (normal versus mild), *p* = 0.1803 (mild versus moderate to severe), *p* = 0.0002 (normal versus moderate to severe), and overall *p* = 0.0019) (Figure 4A).

The percentage of SARC-F score ≥ 4 in categories of normal condition, mild malnutritional condition, and moderate to severe malnutritional condition in patients without advanced cancer were: 4.5% (10/223) in the normal, 10.3% (24/234) in the mild, and 23.3% (21/90) in the moderate to severe, respectively, (*p* = 0.0206 (normal versus mild), *p* = 0.0038 (mild versus moderate to severe), *p* < 0.0001 (normal versus moderate to severe), and overall *p* < 0.0001). (Figure 4B).

### 3.6. SARC-F Score Based on the Nutritional Condition Stratified by the Anatomical Categories of Disease

**Figure 5:**
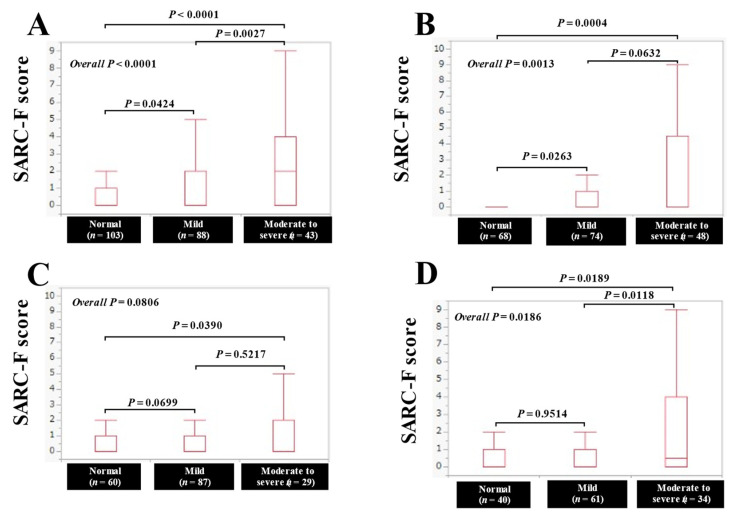
The SARC-F score based on the nutritional condition stratified by the anatomical categories of disease. (**A**) upper gastrointestinal disease (UGD). (**B**) lower gastrointestinal disease (LGD). (**C**) biliary and pancreatic disease (BPD). (**D**) liver disease (LD).

The median (IQR) SARC-F scores in categories of normal condition, mild malnutritional condition, and moderate to severe malnutritional condition in patients with UGD (*n* = 234) were: 0 (0–1) in the normal (*n* = 103), 0 (0–2) in the mild (*n* = 88), and 2 (0–4) in the moderate to severe (*n* = 43), respectively, (*p* = 0.0424 (normal versus mild), *p* = 0.0027 (mild versus moderate to severe), *p* < 0.0001 (normal versus moderate to severe), and overall *p* < 0.0001). (Figure 5A) The median (IQR) SARC-F scores in categories of normal condition, mild malnutritional condition, and moderate to severe malnutritional condition in patients with LGD (*n* = 190) were: 0 (0–0) in the normal (*n* = 68), 0 (0–1) in the mild (*n* = 74), and 0 (0–4) in the moderate to severe (*n* = 48), respectively, (*p* = 0.0263 (normal versus mild), *p* = 0.0632 (mild versus moderate to severe), *p* = 0.0004 (normal versus moderate to severe), and overall *p* = 0.0013). (Figure 5B) The median (IQR) SARC-F scores in categories of normal condition, mild malnutritional condition, and moderate to severe malnutritional condition in patients with BPD (*n* = 176) were: 0 (0–1) in the normal (*n* = 60), 0 (0–1) in the mild (*n* = 87), and 0 (0–2) in the moderate to severe (*n* = 29), respectively, (*p* = 0.0609 (normal versus mild), *p* = 0.5217 (mild versus moderate to severe), *p* = 0.0390 (normal versus moderate to severe), and overall *p* = 0.0806). (Figure 5C) The median (IQR) SARC-F scores in categories of normal condition, mild malnutritional condition, and moderate to severe malnutritional condition in patients with LD (*n* = 135) were: 0 (0–1) in the normal (*n* = 40), 0 (0–1) in the mild (*n* = 61), and 0.5 (0–4) in the moderate to severe (*n* = 34), respectively, (*p* = 0.9514 (normal versus mild), *p* = 0.0118 (mild versus moderate to severe), *p* = 0.0189 (normal versus moderate to severe), and overall *p* = 0.0186) (Figure 5D).

### 3.7. The Percentage of Subjects with SARC-F Score ≥ 4 Based on the Nutritional Condition Stratified by the Anatomical Categories of Disease

**Figure 6:**
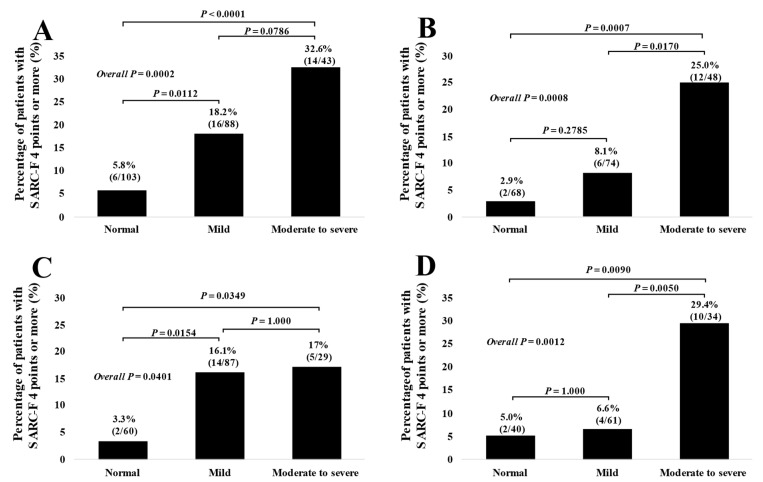
The percentage of patients with SARC-F score ≥ 4 based on the nutritional condition stratified by the anatomical categories of disease. (**A**) upper gastrointestinal disease (UGD). (**B**) lower gastrointestinal disease (LGD). (**C**) biliary and pancreatic disease (BPD). (**D**) liver disease (LD).

The percentage of SARC-F score ≥ 4 in categories of normal condition, mild malnutritional condition, and moderate to severe malnutritional condition in patients with UGD were: 5.8% (6/103) in the normal, 18.2% (16/88) in the mild, and 32.6% (14/43) in the moderate to severe, respectively, (*p* = 0.0112 (normal versus mild), *p* = 0.0786 (mild versus moderate to severe), *p* < 0.0001 (normal versus moderate to severe), and overall *p* = 0.0002). (Figure 6A) The percentage of SARC-F score ≥ 4 in categories of normal condition, mild malnutritional condition, and moderate to severe malnutritional condition in patients with LGD were: 2.9% (2/68) in the normal, 8.1% (6/74) in the mild, and 25.0% (12/48) in the moderate to severe, respectively, (*p* = 0.2785 (normal versus mild), *p* = 0.0170 (mild versus moderate to severe), *p* = 0.0007 (normal versus moderate to severe), and overall *p* = 0.0008). (Figure 6B) The percentage of SARC-F score ≥ 4 in categories of normal condition, mild malnutritional condition, and moderate to severe malnutritional condition in patients with BPD were: 3.3% (2/60) in the normal, 16.1% (14/87) in the mild, and 17.2% (5/29) in the moderate to severe, respectively, (*p* = 0.0154 (normal versus mild), *p* = 1.000 (mild versus moderate to severe), *p* = 0.0349 (normal versus moderate to severe), and overall *p* = 0.0401). (Figure 6C) The percentage of SARC-F score ≥ 4 in categories of normal condition, mild malnutritional condition, and moderate to severe malnutritional condition in patients with LD were: 5.0% (2/40) in the normal, 6.6% (4/61) in the mild, and 29.4% (10/34) in the moderate to severe, respectively, (*p* = 1.000 (normal versus mild), *p* = 0.0050 (mild versus moderate to severe), *p* = 0.0090 (normal versus moderate to severe), and overall *p* = 0.0012). (Figure 6D).

### 3.8. Uni- and Multivariate Analysis of Variables for the CONUT Score ≥ 2 or the CONUT Score ≥ 5

In the univariate analysis of variables for the CONUT score ≥ 2 (mild or more advanced malnutrition), age (*p* = 0.0485), BMI (*p* = 0.020), SARC-F score (*p* < 0.0001), estimated glomerular filtration rate (eGFR, *p* = 0.0020), and C reactive protein (CRP, *p* < 0.0001) were significant factors. The SARC-F score (*p* < 0.0001), BMI (*p* = 0.0107), eGFR (*p* = 0.0044) and CRP (*p* < 0.0001) were independent factors for the CONUT score ≥ 2 in the multivariate analysis (Table 2). Likewise, in the univariate analysis of variables for the CONUT score ≥ 5 (moderate or more advanced malnutrition), the SARC-F score (*p* < 0.0001), eGFR (*p* = 0.0010) and CRP (*p* < 0.0001) were significant factors. The SARC-F score (*p* < 0.0001), eGFR (*p* = 0.0041) and CRP (*p* < 0.0001) were independent factors for the CONUT score ≥ 5 in the multivariate analysis (Table 2).

### 3.9. Multivariate Analysis Using Cumulative Logistic Model

The study cohort can be classified into four groups based on the CONUT score (0–1, 2–4, 5–8, and 9–12). Thus, we further performed multivariate analysis for the CONUT score using the cumulative logistic model. As shown in Table 3, SARC-F score, the SARC-F score, BMI, eGFR and CRP were independent predictors for the CONUT score.

### 3.10. ROC Analysis for the Malnutrition as Evaluated by the CONUT Score

**Figure 7:**
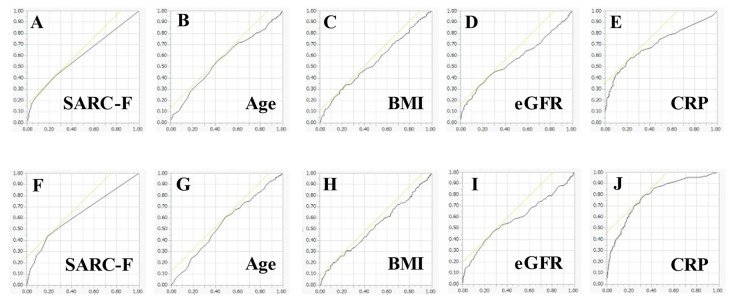
Receiver operating characteristic curves for the CONUT score ≥ 2 (**A**–**E**) and for the CONUT score ≥ 5 (**F**–**J**). The vertical axis indicates sensitivity, and the horizontal axis indicates 1-specificity.

ROC analysis for the malnutrition was performed. In the ROC analysis for the CONUT score ≥ 2, CRP had the highest AUC (AUC = 0.70), followed by the SARC-F score (AUC = 0.60). (Table 3) In the ROC analysis for the CONUT score ≥ 5, CRP had the highest AUC (AUC = 0.79), followed by the SARC-F score (AUC = 0.63). (Table 4 and Figure 7) The sensitivity, specificity and optimal cutoff point in each variable are summarized in Table 3.

### 3.11. ROC Analysis of the CONUT Score for the SARC-F Score 4 or More

**Figure 8:**
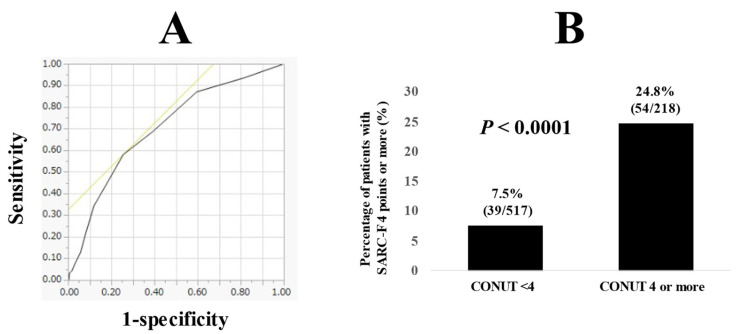
(**A**) ROC curve of the CONUT score for the SARC-F ≥ 4. (**B**) The prevalence of patients with SARC-F ≥ 4 in patients with the CONUT score < 4 (*n* = 517) and CONUT score ≥ 4 (*n* = 218).

ROC analysis of the CONUT score for the SARC-F score 4 or more (highly possibility of sarcopenia) was also performed (Figure 8A). The best cutoff point of the CONUT score was 4. The AUC and sensitivity/specificity was 0.70 and 58.1/74.5%. The prevalence of patients with SARC-F score ≥ 4 in patients with the CONUT score ≥ 4 was significantly higher than that in patients with the CONUT score <4 (24.8% (54/218) vs. 7.5% (39/517), *p* < 0.0001) (Figure 8B).

## 4. Discussion

Sarcopenia research is rapidly advancing as the disease attracts more attention around the world. The development of new drugs such as anti-myostatin antibodies to improve sarcopenia is also underway [27]. As described earlier, SARC-F is recommended to use as a first screening tool for sarcopenia in the current international guidelines [17,20,21]. The SARC-F score is reported to be associated with physical functional reserve and QOL [18,19]. The CONUT score is a well validated screening tool for malnutrition [25,26,28,29,30]. The usefulness of the CONUT score as a prognostic factor in various malignancies has been demonstrated [28,29,31,32,33,34]. A nutritional assessment by the CONUT score is also recommended in patients with COVID-19 [35]. However, to our knowledge, no reports can be found comparing the SARC-F score and the CONUT score in patients with GDs. The current study seems to be the initial effort at clarifying these research questions. A large cohort (*n* = 735) is one of the major strengths of the present study.

Overall, the SARC-F score and the percentage of patients with a SARC-F score ≥ 4 were well stratified by the malnutritional status as assessed by the CONUT score in all analyses. Additionally, the SARC-F score was an independent factor associated with both the CONUT score ≥ 2 and the CONUT score ≥ 5, and in the multivariate analysis using the cumulative logistic model, the SARC-F score was consistently significant for the CONUT score. These observations imply that the SARC-F score accurately reflects the nutritional condition of patients with GDs and sheds some light on the understanding of the relationship between malnutritional status and sarcopenia in patients with GDs. The SARC-F score and the percentage of patients with a SARC-F score ≥ 4 between moderate and severe malnutritional condition were similar, so there is no need to separate the two conditions in light of the incidence of sarcopenia.

In our data, 63.1% (464/735) had mild or more advanced malnutritional status as assessed by the CONUT score at baseline. Considering our data, the evaluation for nutritional status in patients with GDs seems to be mandatory. The percentage of subjects with a SARC-F score ≥ 4 in advanced cancer patients with normal nutritional conditions was almost similar to that in non-advanced patients with normal nutritional conditions (4.2% versus 4.5%). In GD patients with normal nutritional condition, advanced cancer itself may not affect the incidence of sarcopenia. The optimal cutoff points of the SARC-F score for the CONUT score ≥ 2 and ≥5 were 1 and 2 in our ROC analysis. Patients with a SARC-F score of 1 or higher should be evaluated for nutritional status as if they are under malnutritional condition. In addition to blood tests, it is also important to ask the patient if he or she is eating well. The sensitivity and specificity of the SARC-F score for the CONUT score ≥ 2 were 42.0% and 74.5%, whereas those for the CONUT score ≥ 5 were 43.5% and 81.4%. As mentioned earlier, relatively lower sensitivity of the SARC-F score for sarcopenia may be a concern, which is similar to our results [22,23,24]. To the contrary, the optimal cutoff point of the CONUT score for the SARC-F ≥4 (highly possibility for sarcopenia) was 4 points. In patients with a CONUT score ≥ 4, evaluation of muscle strength and muscle mass should be considered.

In the ROC analyses both for mild or more severe malnutrition and for the moderate or more severe malnutrition, CRP had the highest AUC. Proinflammatory cytokines include IL-1β, IL-6, TNF-α, and IFN-γ, and they cause decreased appetite and increased energy expenditure, which can be related to our results [36]. Cachexia is a common form of disease-related malnutrition associated with inflammation and loss of skeletal muscle mass. Although cachexia is common in the daily clinical practice of patients with chronic wasting or inflammatory diseases, it is not fully recognized by medical professionals, and many cases of undiagnosed cachexia result in further worsening nutritional status and loss of skeletal muscle mass and physical function [37,38]. Clinicians should be fully aware of these. In our ROC analysis, the AUC of SARC-F for the mild or more severe malnutrition and for the moderate or more severe malnutrition was higher than that of BMI, which is a good indicator of nutritional status [39]. The SARC-F score rather than BMI can be a good indicator for malnutrition. We would like to emphasize this point. eGFR, on the other hand, was an independent variable associated with both the CONUT score ≥ 2 and ≥5 in our multivariate analysis. Intravascular dehydration due to malnutrition may be related to the present results [40].

Several limitations must be acknowledged in the current analysis. First, the current study was a cross-sectional study at a single hospital with a retrospective nature. Second, the exact number of patients with a definite diagnosis of sarcopenia was unclear in our data. Third, our study cohort was highly heterogeneous including broad spectrum of GDs. Nevertheless, our study results indicated that although CRP had the highest AUC for the malnutrition, the SARC-F score can correlate with the CONUT score in patients with GDs as well as CRP.

In conclusion, the SARC-F score in patients with GDs can reflect malnutritional status. The SARC-F score can be associated with various clinical parameters in patients with GDs.

## Figures and Tables

**Table 1:** Baseline characteristics (*n* = 735).

	*n* or Median (IQR)
Age (years)	71 (62–77)
Gender, male/female	438/297
Type of disease	
Upper gastrointestinal disease	234
Lower gastrointestinal disease	190
Biliary and pancreatic disease	176
Liver disease	135
Advanced cancer, yes	188
Body mass index (kg/m^2^)	22.2 (19.7–24.5)
C reactive protein (mg/dL)	0.22 (0.06–1.21)
eGFR (ml/min/1.73 m^2^)	68 (55–81)
Serum albumin	
≥3.5 g/dL	534
≥3.0 g/dL, <3.5 g/dL	116
≥2.5 g/dL, <3.0 g/dL	54
<2.5 g/dL	31
Total cholesterol	
≥180 mg/dL	375
≥140 mg/dL, <180 mg/dL	243
≥100 mg/dL, <140 mg/dL	98
<100 mg/dL	19
Total lymphocyte count	
≥1600/μL	232
≥1200/μL, <1600/μL	191
≥800/μL, <1200/μL	185
<800/μL	127
CONUT score	
Normal (0 or 1 point)	271
Mild malnutrition (2–4 points)	310
Moderate malnutrition (5–8 points)	127
Severe malnutrition (9–12 points)	27

IQR; interquartile range, eGFR; estimated glomerular filtration rate, CONUT; Controlling Nutritional Status.

**Table 2:** Univariate and multivariate analyses of factors associated with the CONUT score ≥ 2 or the CONUT score ≥ 5.

The CONUT Score ≥ 2	Univariate	Multivariate		
	*p* Value	OR	95% CI	*p* Value
SARC-F score (per one)	<0.0001	1.233	[1.109, 1.372]	<0.0001
Age (per one year)	0.0485	1.007	[0.994, 1.020]	0.3256
BMI (per one kg/m^2^)	0.020	0.948	[0.910, 0.988]	0.0107
eGFR (per one ml/min/1.73 m^2^)	0.002	0.988	[0.980, 0.997]	0.0044
CRP (per one mg/dl)	<0.0001	1.845	[1.508, 2.257]	<0.0001
**The CONUT Score ≥ 5**	**Univariate**	**Multivariate**		
	***p*** **Value**	**OR**	**95% CI**	***p*** **Value**
SARC-F score (per one)	<0.0001	1.233	[1.134, 1.341]	<0.0001
Age (per one year)	0.0908			
BMI (per one kg/m^2^)	0.5227			
eGFR (per one ml/min/1.73 m^2^)	0.001	0.988	[0.979, 0.996]	0.0041
CRP (per one mg/dl)	<0.0001	1.260	[1.184, 1.341]	<0.0001

CONUT; Controlling Nutritional Status, BMI; body mass index, eGFR; estimated glomerular filtration rate, CRP; C reactive protein, OR; odds ratio, CI; confidence interval.

**Table 3:** Multivariate analysis using cumulative logistic model for the CONUT score.

	Estimates	Standard Error	*p* Value	95% CI
SARC-F score	−0.266	0.0989	0.0071	[−0.456, −0.065]
Age	−0.0128	0.0202	0.5250	[−0.055, 0.0244]
BMI	0.1196	0.0608	0.0491	[0.004, 0.242]
eGFR	0.0218	0.0101	0.0312	[0.002, 0.042]
CRP	−0.8115	0.1089	<0.0001	[−1.041, −0.613]

BMI; body mass index, eGFR; estimated glomerular filtration rate, CRP; C reactive protein, OR; odds ratio, CI; confidence interval.

**Table 4:** ROC analysis for the CONUT score ≥ 2 or ≥5.

The CONUT Score ≥ 2	AUC	Sensitivity (%)	Specificity (%)	Cutoff Point
SARC-F score	0.60	42.0	74.5	1
Age (year)	0.57	56.5	56.8	71
BMI (kg/m^2^)	0.55	33.8	67.1	20.2
eGFR (ml/min/1.73 m^2^)	0.57	32.8	83.4	58
CRP (mg/dl)	0.70	57.8	77.5	0.27
**The CONUT Score ≥ 5**	**AUC**	**Sensitivity (%)**	**Specificity (%)**	**Cutoff Point**
SARC-F score	0.63	43.5	81.4	2
Age (year)	0.55	61.0	51.0	71
BMI (kg/m^2^)	0.52	20.3	87.6	18.3
eGFR (ml/min/1.73 m^2^)	0.59	47.1	72.2	55
CRP (mg/dl)	0.79	77.9	68.2	0.32

CONUT; Controlling Nutritional Status, BMI; body mass index, eGFR; estimated glomerular filtration rate, CRP; C reactive protein, AUC; area under the receiver operating characteristics curve.

## Data Availability

The data presented in this study are available on request from the corresponding author. The data are not publicly available due to personal information.

## Supplementary Materials

The following supporting information can be downloaded at: https://www.mdpi.com/article/10.3390/jcm11030582/s1, Table S1: Details of diagnosis names (*n* = 735).
