# Supplementary material for: The Relationship between the SARC-F Score and the Controlling Nutritional Status Score in Gastrointestinal Diseases
Source: J Clin Med. 2022 Jan 24;11(3):582. doi: 10.3390/jcm11030582 (PMC8836691; doi:10.3390/jcm11030582)
Supplement: Supplementary file 1 [file jcm-11-00582-s001.zip › jcm-1536275-supplementary.pdf]

Table S1. Details of diagnosis names (n=735).

Upper gastrointestinal disease (n=234)	
Disease name	Patient number
Advanced esophageal cancer	35
Advanced gastric cancer	31
Early esophageal cancer	26
Early gastric cancer	70
Early duodenal cancer	3
Upper gastrointestinal bleeding	19
Esophageal varices	15
Gastroesophageal reflux disease	9
Esophageal benign polyp	1
Gastric benign polyp	14
Duodenal benign polyp	5
Others	6

Lower gastrointestinal disease (n=190)	
Disease name	Patient number
Advanced colorectal cancer	40
Advanced small intestine cancer	1
Early colorectal cancer	19
Colorectal benign polyp	52
Crohn disease (CD)	22
Ulcerative colitis (UC)	22
Colitis (other than CD or UC)	9
Lower gastrointestinal bleeding	13
Ileus	5
Others	7

Biliary and pancreatic disease (n=176)	
Disease name	Patient number
Advanced pancreatic cancer	33
Advanced gallbladder cancer	3
Advanced biliary tract cancer	10

Early pancreatic cancer	19
Early biliary tract cancer	1
Acute cholangitis	15
Common bile duct stone	14
Acute cholecystitis	12
Acute pancreatitis	8
Chronic pancreatitis	12
Pancreatic benign tumor	23
Duodenal benign tumor	1
Obstructive jaundice	9
Autoimmune pancreatitis	2
Primary sclerosing cholangitis	2
Carcinoma of the Ampulla of Vater	3
Biliary tract stenosis	6
Others	3

Liver disease (n=135)	
Disease name	Patient number
Advanced hepatocellular carcinoma	31
Advanced intrahepatic cholangiocarcinoma	4
Acute liver injury	18
Chronic hepatitis	6
Early hepatocellular carcinoma	56
Early intrahepatic cholangiocarcinoma	4
Intrahepatic biliary tract stone	3
Refractory ascites	3
Portal vein thrombosis	2
Liver abcess	1
Liver failure	1
Wilson disease	1
Primary biliary cholangitis	1
Others	4
